# The evolution of symptom burden in older men and women with advanced CKD

**DOI:** 10.1093/ndt/gfag045

**Published:** 2026-03-18

**Authors:** Nicholas C Chesnaye, Jenny Wrackefeldt, Fergus J Caskey, Friedo W Dekker, Claudia Torino, Maciej Szymczak, Christiane Drechsler, Christoph Wanner, Antonio Vilasi, Yvette Meuleman, Maria Pippias, Vianda S Stel, Kitty J Jager, Marie Evans, the EQUAL study investigators, Andreas Schneider, Anke Torp, Beate Iwig, Boris Perras, Christian Marx, Christiane Drechsler, Christof Blaser, Christoph Wanner, Claudia Emde, Detlef Krieter, Dunja Fuchs, Ellen Irmler, Eva Platen, Hans Schmidt-Gürtler, Hendrik Schlee, Holger Naujoks, Ines Schlee, Sabine Cäsar, Joachim Beige, Jochen Röthele, Justyna Mazur, Kai Hahn, Katja Blouin, Katrin Neumeier, Kirsten Anding-Rost, Lothar Schramm, Monika Hopf, Nadja Wuttke, Nikolaus Frischmuth, Pawlos Ichtiaris, Petra Kirste, Petra Schulz, Sabine Aign, Sandra Biribauer, Sherin Manan, Silke Röser, Stefan Heidenreich, Stephanie Palm, Susanne Schwedler, Sylke Delrieux, Sylvia Renker, Sylvia Schättel, Theresa Stephan, Thomas Schmiedeke, Thomas Weinreich, Til Leimbach, Torsten Stövesand, Udo Bahner, Wolfgang Seeger, Adamasco Cupisti, Adelia Sagliocca, Alberto Ferraro, Alessandra Mele, Alessandro Naticchia, Alex Còsaro, Andrea Ranghino, Andrea Stucchi, Angelo Pignataro, Antonella De Blasio, Antonello Pani, Aris Tsalouichos, Bellasi Antonio, Biagio Raffaele Di Iorio, Butti Alessandra, Cataldo Abaterusso, Chiara Somma, Claudia D’alessandro, Claudia Torino, Claudia Zullo, Claudio Pozzi, Daniela Bergamo, Daniele Ciurlino, Daria Motta, Domenico Russo, Enrico Favaro, Federica Vigotti, Ferruccio Ansali, Ferruccio Conte, Francesca Cianciotta, Francesca Giacchino, Francesco Cappellaio, Francesco Pizzarelli, Gaetano Greco, Gaetana Porto, Giada Bigatti, Giancarlo Marinangeli, Gianfranca Cabiddu, Giordano Fumagalli, Giorgia Caloro, Giorgina Piccoli, Giovanbattista Capasso, Giovanni Gambaro, Giuliana Tognarelli, Giuseppe Bonforte, Giuseppe Conte, Giuseppe Toscano, Goffredo Del Rosso, Irene Capizzi, Ivano Baragetti, Lamberto Oldrizzi, Loreto Gesualdo, Luigi Biancone, Manuela Magnano, Marco Ricardi, Maria Di Bari, Maria Laudato, Maria Luisa Sirico, Martina Ferraresi, Michele Provenzano, Moreno Malaguti, Nicola Palmieri, Paola Murrone, Pietro Cirillo, Pietro Dattolo, Pina Acampora, Rita Nigro, Roberto Boero, Roberto Scarpioni, Rosa Sicoli, Rosella Malandra, Silvana Savoldi, Silvio Bertoli, Silvio Borrelli, Stefania Maxia, Stefano Maffei, Stefano Mangano, Teresa Cicchetti, Tiziana Rappa, Valentina Palazzo, Simone De Walter, Schrander Anita, Bastiaan van Dam, Carl Siegert, Carlo Gaillard, Charles Beerenhout, Cornelis Verburgh, Cynthia Janmaat, Ellen Hoogeveen, Ewout Hoorn, Friedo Dekker, Johannes Boots, Henk Boom, Jan-Willem Eijgenraam, Jeroen Kooman, Joris Rotmans, Kitty Jager, Liffert Vogt, Maarten Raasveld, Marc Vervloet, Marjolijnvan Buren, Merelvan Diepen, Nicholas Chesnaye, Paul Leurs, Pauline Voskamp, Peter Blankestijn, Sadie van Esch, Siska Boorsma, Stefan Berger, Constantijn Konings, Zeynep Aydin, Aleksandra Musiała, Anna Szymczak, Ewelina Olczyk, Hanna Augustyniak-Bartosik, Ilona Miskowiec-Wisniewska, Jacek Manitius, Joanna Pondel, Kamila Jedrzejak, Katarzyna Nowanska, Łukasz Nowak, Maciej Szymczak, Magdalena Durlik, Szyszkowska Dorota, Teresa Nieszporek, Zbigniew Heleniak, Andreas Jonsson, Björn Rogland, Carin Wallquist, Denes Vargas, Emöke Dimény, Fredrik Sundelin, Fredrik Uhlin, Gunilla Welander, Isabel Bascaran Hernandez, Knut-Christian Gröntoft, Maria Stendahl, Maria Svensson, Marie Evans, Olof Heimburger, Pavlos Kashioulis, Stefan Melander, Tora Almquist, Alistair Woodman, Anna McKeever, Asad Ullah, Barbara McLaren, Camille Harron, Carla Barrett, Charlotte O’Toole, Christina Summersgill, Colin Geddes, Deborah Glowski, Deborah McGlynn, Dympna Sands, Fergus Caskey, Geena Roy, Gillian Hirst, Hayley King, Helen McNally, Houda Masri-Senghor, Hugh Murtagh, Hugh Rayner, Jane Turner, Joanne Wilcox, Jocelyn Berdeprado, Jonathan Wong, Joyce Banda, Kirsteen Jones, Lesley Haydock, Lily Wilkinson, Margaret Carmody, Maria Weetman, Martin Joinson, Mary Dutton, Michael Matthews, Neal Morgan, Nina Bleakley, Paul Cockwell, Paul Roderick, Phil Mason, Philip Kalra, Rincy Sajith, Sally Chapman, Santee Navjee, Sarah Crosbie, Sharon Brown, Sheila Tickle, Suresh Mathavakkannan, Ying Kuan

**Affiliations:** ERA Registry, Department of Medical Informatics, Amsterdam UMC, University of Amsterdam, DE Amsterdam, Amsterdam, the Netherlands; Amsterdam Public Health Research Institute, Quality of Care, Amsterdam, the Netherlands; Renal Unit, Department of Clinical Intervention and Technology (CLINTEC), Karolinska Institutet and Karolinska University Hospital, Stockholm, Sweden; Population Health Sciences, Bristol Medical School, University of Bristol, Bristol, UK; Department of Clinical Epidemiology, Leiden University Medical Center, RC Leiden, Leiden, the Netherlands; IFC-CNR, Clinical Epidemiology and Pathophysiology of Renal Diseases and Hypertension, Reggio Calabria, Italy; Dept of Nephrology and Transplantation Medicine, Wroclaw Medical University, Wroclaw, Poland; Department of Clinical Research and Epidemiology, Comprehensive Heart Failure Center, University Hospital of Würzburg, Würzburg, Germany; Department of Clinical Research and Epidemiology, Comprehensive Heart Failure Center, University Hospital of Würzburg, Würzburg, Germany; IFC-CNR, Clinical Epidemiology and Pathophysiology of Renal Diseases and Hypertension, Reggio Calabria, Italy; Department of Clinical Epidemiology, Leiden University Medical Center, RC Leiden, Leiden, the Netherlands; Population Health Sciences, Bristol Medical School, University of Bristol, Bristol, UK; North Bristol NHS Trust, Renal Unit, Bristol Medical School, Bristol, UK; ERA Registry, Department of Medical Informatics, Amsterdam UMC, University of Amsterdam, DE Amsterdam, Amsterdam, the Netherlands; Amsterdam Public Health Research Institute, Quality of Care,Amsterdam, the Netherlands; ERA Registry, Department of Medical Informatics, Amsterdam UMC, University of Amsterdam, DE Amsterdam, Amsterdam, the Netherlands; Amsterdam Public Health Research Institute, Quality of Care,Amsterdam, the Netherlands; Renal Unit, Department of Clinical Intervention and Technology (CLINTEC), Karolinska Institutet and Karolinska University Hospital, Stockholm, Sweden

**Keywords:** chronic kidney disease, gender, kidney failure, patient-reported outcomes, symptom burden

## Abstract

**Background and hypothesis:**

Whether the interdependence of symptom burden and kidney function over time differ by gender remain unknown. Here we aim to describe longitudinal trajectories of symptom burden in older men and women with advanced chronic kidney disease (CKD) over time, and in relation to the trajectory of kidney function.

**Methods:**

In the prospective, observational multicentre cohort study in Europe; The European QUALity (EQUAL) study on treatment in advanced CKD, patients with incident eGFR <20 ml/min per 1.73 m^2^ not on dialysis, ≥65 years of age were followed for over 5 years, or until initiation of kidney replacement therapy. Symptom number and burden were assessed at 3–6-month intervals using the dialysis symptoms index. The evolution of symptom burden was modelled over time and eGFR for men and women separately, using generalized additive models.

**Results:**

Over the follow-up, 4730 symptom measurements were collected in 1135 patients. Overall symptom progression was relatively slow. The number of symptoms increased on average by 0.47 (95% CI 0.37–0.58) symptoms per year, and by 0.75 (95% CI 0.58–0.91) symptoms per 5 ml/min/1.73 m^2^ decrease in kidney function. Women reported more symptoms on average than men throughout follow-up, although the increase in symptom number with declining kidney function was almost three times greater in men (0.97 per 5 ml/min/1.73 m^2^, 95% CI 0.78–1.16) than in women (0.34 per 5 ml/min/1.73 m^2^, 95% CI 0.03–0.64). This difference was especially evident towards lower eGFR levels.

**Conclusions:**

Women experience more symptoms on average than men, but symptoms progressed faster in men, relative to kidney decline. Further research should focus on how patient-reported outcome measures (PROMs) can routinely be implemented in nephrology care, and how to use PROMs effectively in patient–nephrologist communication with regards to shared decision making.

## Graphical Abstract

**Graphical Abstract:**
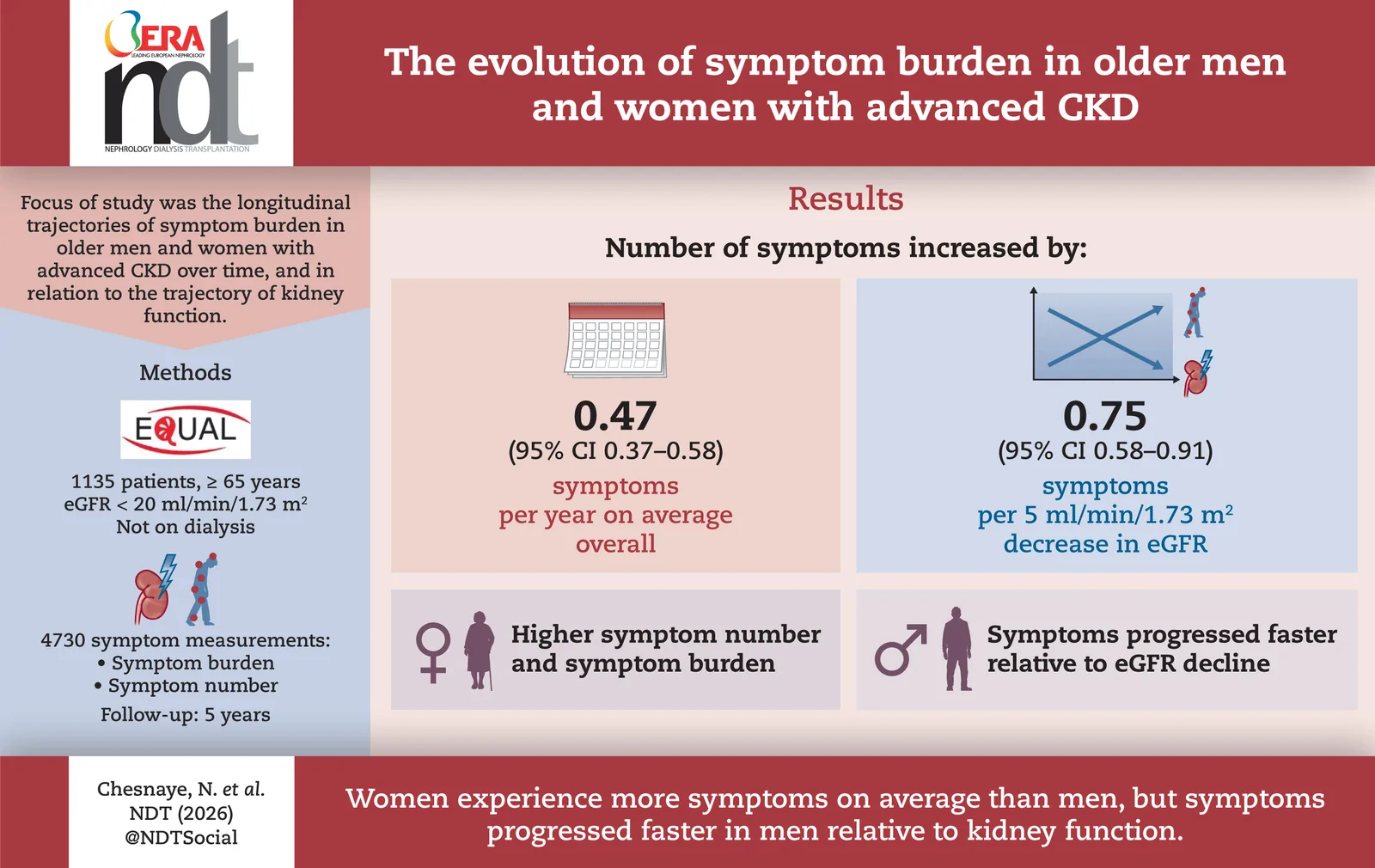

KEY LEARNING POINTS **What was known:**

- CKD patients with a higher symptom burden display lower quality of life.
- The symptom burden is substantial already at moderately reduced kidney function with a higher burden in women compared with men.
- There is conflicting evidence of the association between symptoms and estimated glomerular filtration rate and a lack of prospective studies of symptom trajectories in men and women with advanced stage CKD.

**This study adds:**

- Overall symptom progression was relatively slow and nonlinear, both over time and over eGFR decline; symptom burden rose especially when eGFR dropped below 10 ml/min/1.73 m^2^.
- Symptoms increased in both men and women and displayed different trajectories for different symptoms.
- Despite women reporting an overall higher symptom burden, the increase in symptom burden over time was twice as fast in men during follow-up, and almost three times faster in men when evaluated over eGFR decline.

**Potential impact:**

- Recognizing uremic symptoms in non-dialysis CKD patients opens up for earlier disease management.
- Measuring symptoms using PROMs in routine nephrology outpatient care may help personalize treatment approaches.

## INTRODUCTION

Patient-reported outcome measures (PROMs) are widely used to assess patients’ perspectives on their well-being. Often they involve questions about patient-reported outcomes, the ability to perform daily activities, and health-related quality of life [1]. Importantly, PROMs results offer a more holistic view of a patient’s health beyond clinical outcomes, improving communication with patients. During the Kidney Disease: Improving Global Outcomes (KDIGO) controversies conference in 2018, one of the key messages conveyed was the need to better involve patients’ perspectives such as PROMs results into clinical decision making [2].

When patients with chronic kidney disease (CKD) are asked about their top research priorities, the PROM considering symptoms such as fatigue, anxiety, sleep quality, and restless legs rated among those most important [3]. Despite this, the recognition and management of symptoms in individuals with CKD remains inadequate across the continuum of care. Evidence indicates that healthcare professionals frequently underestimate the symptom burden experienced by their patients, especially in non-dialysis patients, leading to underdiagnosis and undertreatment of distressing symptoms. A large multicentre study from the Chronic Kidney Disease Outcome Patterns Study (CKDopps) recently demonstrated a significant symptom burden among patients with a mean estimated glomerular filtration rate (eGFR) of 30 ml/min/1.73 m^2^, with a higher symptom burden in women as compared to men [4]. But, despite our general understanding that symptom burden increases as CKD progresses [5], the CKDopps study did not find a higher symptom burden in patients with lower eGFR [4]. However, the study was limited by a cross-sectional design, and there are few, if any, studies exploring temporal changes in symptom burden in patients with severely decreased eGFR not on dialysis, especially those comparing men and women.

Consequently, the interdependence of symptom burden, gender, and kidney function over time remains unknown. In addition, the extent to which individual symptoms contribute to the overall symptom burden, and when these symptoms develop in relation to eGFR has rarely been described. Such research would not only enhance clinical awareness and prioritization of symptom management but also support the integration of PROMs into routine nephrology care. Addressing the symptom burden in CKD non-dialysis represents a critical step toward improving patient-centred outcomes and aligning CKD care with broader goals of quality, equity, and patient empowerment. In the current study, we therefore aim to describe longitudinal trajectories of overall symptom burden and individual symptoms in older men and women with advanced CKD over time, and in relation to the trajectory of kidney function.

## MATERIALS AND METHODS

### Study design and population

The European QUALity Study on treatment in advanced CKD (EQUAL) is an observational multicentre cohort study including CKD patients of 65 years of age and older, with an incident eGFR <20 ml/min/1.73 m² not on dialysis, receiving routine medical care in Germany, Italy, the Netherlands, Poland, Sweden, and the UK. Participants were excluded if the drop in eGFR resulted from an acute event or if they had previously received dialysis or a kidney transplant. Study visits and data collection were scheduled at 3–6-month intervals, and participants were followed for 5 years or until death, dialysis initiation, refusal for further participation, or loss to follow-up. Approval was obtained from the medical ethical committees in each country. Informed consent was obtained from all participants. A full description of the study has been published elsewhere [6]. For the current study, we selected EQUAL participants with available symptoms measurements. A flow chart is presented in Fig. 1.

**Figure 1:**
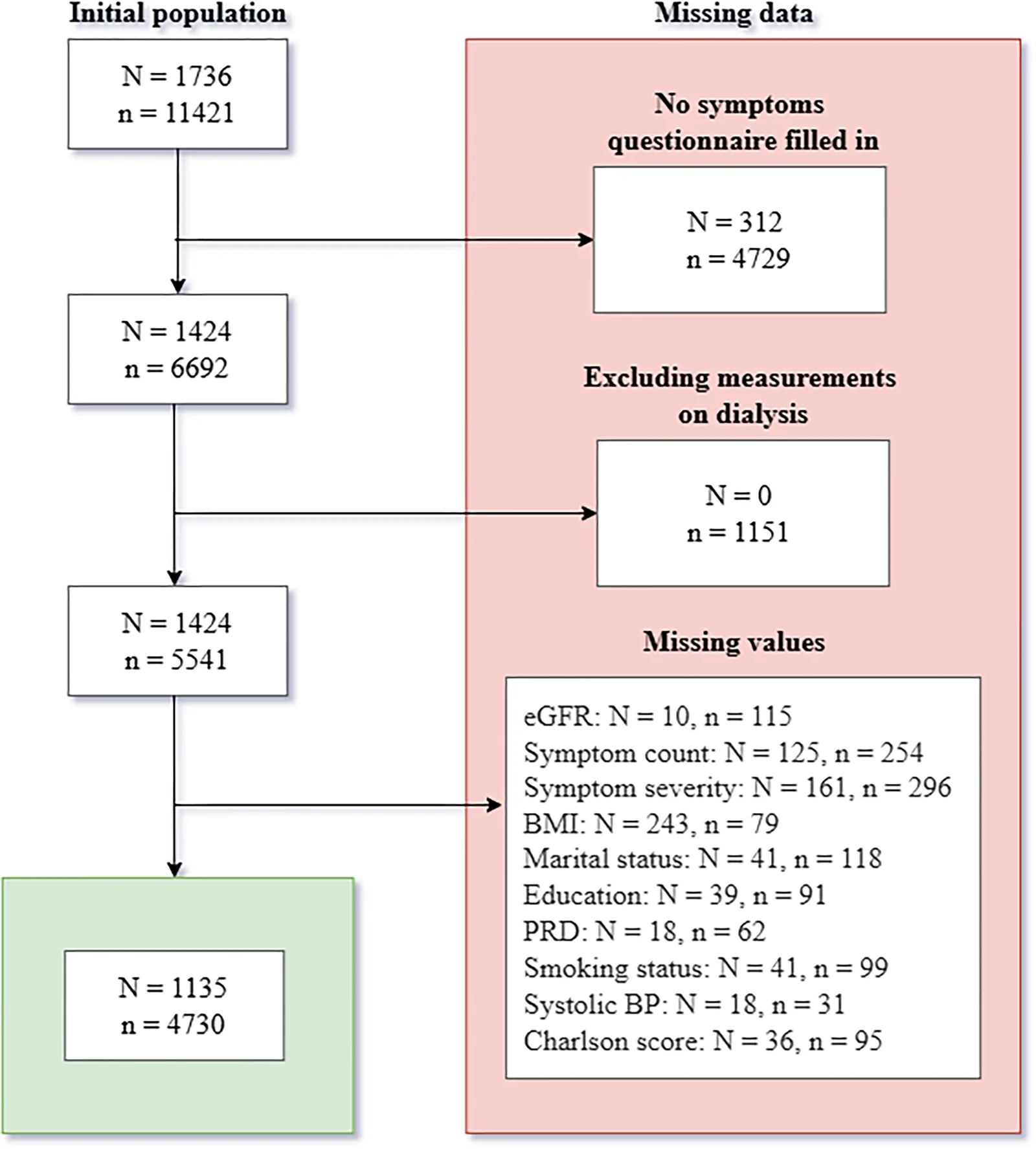
Flow chart. *N* denotes the total number of patients, and *n* refers to the total number of individual study visits.

### Data collection

Clinical data were collected on demographics, primary kidney disease, laboratory data, medication, nutritional status, lifestyle, comorbid conditions, and symptom burden. Gender was self-defined by participants into a binary variable (woman/man). eGFR was calculated from serum creatinine level standardized to isotope dilution mass spectrometry using the 2009 CKD-EPI equation. Primary kidney disease was classified using the codes of the European Renal Association [7].

Symptom burden was measured using self-administered paper questionnaires. Patients reported the presence of symptoms in the past month using the Dialysis Symptom Index, which assesses 30 common symptoms experienced by patients [8]. For each symptom present, patients also rated symptom severity on a five-point Likert scale. We report the total number of symptoms, ranging from 0 to 30, and the total symptom severity score, ranging from 0 to 150. We also report the prevalence of individual symptoms.

### Statistical analysis

The evolution of symptom number and burden, as well as the prevalence of individual symptoms, was modelled over time and over eGFR using generalized additive models (GAM), using a Poisson distribution to model symptom number and symptom severity, and the binomial distribution for the binary variable of symptom prevalence. GAMs do not assume any specific functional form and can be used to reveal nonlinear effects using splines [9]. We used thin-plate regression splines, which place a knot at each unique time value, thus avoiding having to choose an appropriate number and placement of knots [10]. The smoothing parameter (i.e. the amount of ‘wiggliness’) was also determined by the model using restricted maximum likelihood [11]. A random intercept was included to account for the correlation among the repeated measurements taken on the same individual. The analyses were stratified by gender. As for nonlinear models, the slope varies depending on the values of eGFR, we calculated the slope as the derivative, by gender, using a central difference approximation with a small step size. We then averaged the resulting slopes to provide an overall estimate of the effect of eGFR on symptoms progression by gender. To explain any potential differences in the effect of eGFR on symptom burden between men and women, we repeated the analyses with various levels of adjustment. Model 1 adjusts for patient age, marital status, educational class, primary kidney disease (PKD), and country. Model 2 adjusts for the variables listed in model 1, as well as smoking status, blood pressure, and BMI. Model 3 adjusts for the variables listed in model 2, as well as the Charlson comorbidity score. As gender has no true confounders, adjustment variables should be viewed as intermediates in the relationship between gender and symptom burden. The adjustments were performed using inverse probability of treatment weights (IPTW), ensuring a comparable covariate profile in both men and women. Recognizing that symptom burden is linked to death and dialysis initiation that could lead to informative dropout, we addressed potential informative censoring using inverse probability of censoring weights (IPCW). Specifically, we estimated both IPTW and IPCW at each time point using Cox survival models that included the adjustment covariates [12]. Finally, we fitted a marginal structural model by weighting our GAM at each time point with the product of the IPTWs and IPCWs. Missing measurements (Fig. 1) were dealt with through pairwise deletion [13]. All analyses were performed in R version 4.0.2 (R Core Team, Vienna, Austria).

## RESULTS

### Participant characteristics at inclusion

Table 1 describes the baseline characteristics of the 1135 patients by gender. On average, patients were 76 years old at inclusion, two-thirds were men, and the eGFR at baseline was 17.1 ml/min per 1.73 m^2^. Women were slightly older, were more likely to be widowed, had lower levels of education, were less likely to have a history of smoking, had a higher body mass index, and had higher eGFR levels at baseline. Women were less likely to have pre-existing diabetes, coronary artery disease, myocardial infarction, peripheral vascular disease, and malignancies, but more likely to have a history of psychiatric disease. On average, women reported a higher number of symptoms than men (13.6 vs. 10.9) at baseline, as well as a higher symptom severity (40.6 vs. 30.5). During follow-up, 328 (42.3%) of men and 97 (26.9%) of women started dialysis, and 319 (41.1%) of men and 112 (31.1%) of women died.

**Table 1:** Clinical characteristics at inclusion for men and women in the EQUAL Study.

	Overall (*n* = 1135)	Men (*n* = 775)	Women (*n* = 360)	*P*
Age	76.0 (6.6)	75.7 (6.3)	76.6 (7.0)	.036
Marital status				
Married	753 (66.3)	597 (77.0)	156 (43.3)	<.001
Divorced	78 (6.9)	49 (6.3)	29 (8.1)	
Widowed	261 (23.0)	107 (13.8)	154 (42.8)	
Never married	43 (3.8)	22 (2.8)	21 (5.8)	
Education				
None/low	330 (29.1)	197 (25.4)	133 (36.9)	<.001
Intermediate	573 (50.5)	398 (51.4)	175 (48.6)	
High	180 (15.9)	146 (18.8)	34 (9.4)	
Other	52 (4.6)	34 (4.4)	18 (5.0)	
Smoking status				
Current smoker	106 (9.3)	73 (9.4)	33 (9.2)	<.001
Ex-smoker	610 (53.7)	494 (63.7)	116 (32.2)	
No	419 (36.9)	208 (26.8)	211 (58.6)	
Primary renal disease				
Glomerular disease	116 (10.2)	87 (11.2)	29 (8.1)	.007
Tubulo-interstitial disease	104 (9.2)	60 (7.7)	44 (12.2)	
Diabetes	213 (18.8)	161 (20.8)	52 (14.4)	
Hypertension	424 (37.4)	281 (36.3)	143 (39.7)	
Miscellaneous renal disorders	278 (24.5)	186 (24.0)	92 (25.6)	
BMI (kg/m^2^)	28.2 (5.4)	27.9 (5.0)	28.8 (6.1)	.007
Systolic blood pressure (mmHg)	142.4 (21.5)	143.3 (21.2)	140.4 (22.1)	.032
Diastolic blood pressure (mmHg)	74.1 (11.3)	74.5 (11.4)	73.4 (10.9)	.111
Hypertension	992 (89.4)	683 (90.0)	309 (88.3)	.452
Diabetes mellitus	460 (40.6)	337 (43.5)	123 (34.3)	.004
Coronary artery disease	313 (28.2)	248 (32.7)	65 (18.5)	<.001
Myocardial infarction	209 (18.4)	166 (21.4)	43 (11.9)	<.001
Chronic heart failure	209 (18.9)	147 (19.5)	62 (17.5)	.475
Atrial fibrillation	202 (18.0)	137 (17.9)	65 (18.3)	.929
Peripheral vascular disease	197 (17.7)	151 (19.8)	46 (13.0)	0.006
Cerebrovascular disease	169 (15.0)	120 (15.5)	49 (13.7)	.480
Psychiatric disorders	77 (6.8)	39 (5.1)	38 (10.6)	.001
Malignancy	242 (21.5)	185 (24.2)	57 (16.0)	.002
Charlson comorbidity score	7.0 [6.0, 8.0]	7.0 [6.0, 8.0]	7.0 [6.0, 8.0]	.001
CKD-EPI eGFR (ml/min/1.73 m^2^)	17.1 (5.0)	16.8 (4.8)	18.0 (5.3)	<.001
Creatinine (µmol/l)	294.2 (93.8)	320.0 (91.8)	238.9 (71.7)	<.001
Urea (mmol/l)	20.4 (7.8)	20.9 (7.5)	19.4 (8.4)	.002
Sodium (mmol/l)	140.2 (3.2)	140.2 (3.1)	140.1 (3.4)	.655
Potassium (mmol/l)	4.6 (0.6)	4.6 (0.6)	4.5 (0.6)	.009
Calcium (mmol/l)	2.3 (0.2)	2.3 (0.2)	2.3 (0.2)	<.001
Phosphate (mmol/l)	1.3 (0.3)	1.3 (0.3)	1.3 (0.3)	.760
Albumin (g/l)	38.0 (4.9)	38.0 (4.9)	38.0 (4.9)	.894
Hb (g/dl)	11.6 (1.5)	11.7 (1.5)	11.5 (1.4)	.080
Symptom number (0–30)	11.8 (6.0)	10.9 (5.8)	13.6 (5.9)	<.001
Symptom burden (0–150)	33.7 (20.5)	30.5 (19.3)	40.6 (21.4)	<.001

Continuous variables are expressed as mean (SD) except for ACR, and the Charlson comorbidity index, which are presented as median [interquartile range]. Categorical variables are expressed as *n* (%). BMI, body mass index; Hb, haemoglobin; ACR, albumin-creatinine ratio.

### The evolution of symptom number and severity over time

During follow-up, we included 4730 symptoms measurements over a total of 1897 person-years, with a median of 4 (IQR 2–6) measurements per patient and a median follow-up time of 18 months (IQR 6–30). Overall, the number of symptoms increased on average by 0.47 (95% CI 0.37–0.58) symptoms per year, and mean symptom severity increased by 1.57 (95% CI 1.38–1.75) points per year. Despite women reporting an overall higher symptom number throughout follow-up (Fig. 2), the average rate of increase in symptom number was more than twice as fast in men (0.58 per year, 95% CI 0.45–0.71) as in women (0.24 per year, 95% CI 0.05–0.44). Similar results were found for symptom severity (men: 1.96 points per year, 95% CI 1.71–2.21, women: 0.64 points per year, 95% CI 0.29–0.99). Adjustment had little effect on the difference in rate of increase between men and women (Table 2).

**Figure 2:**
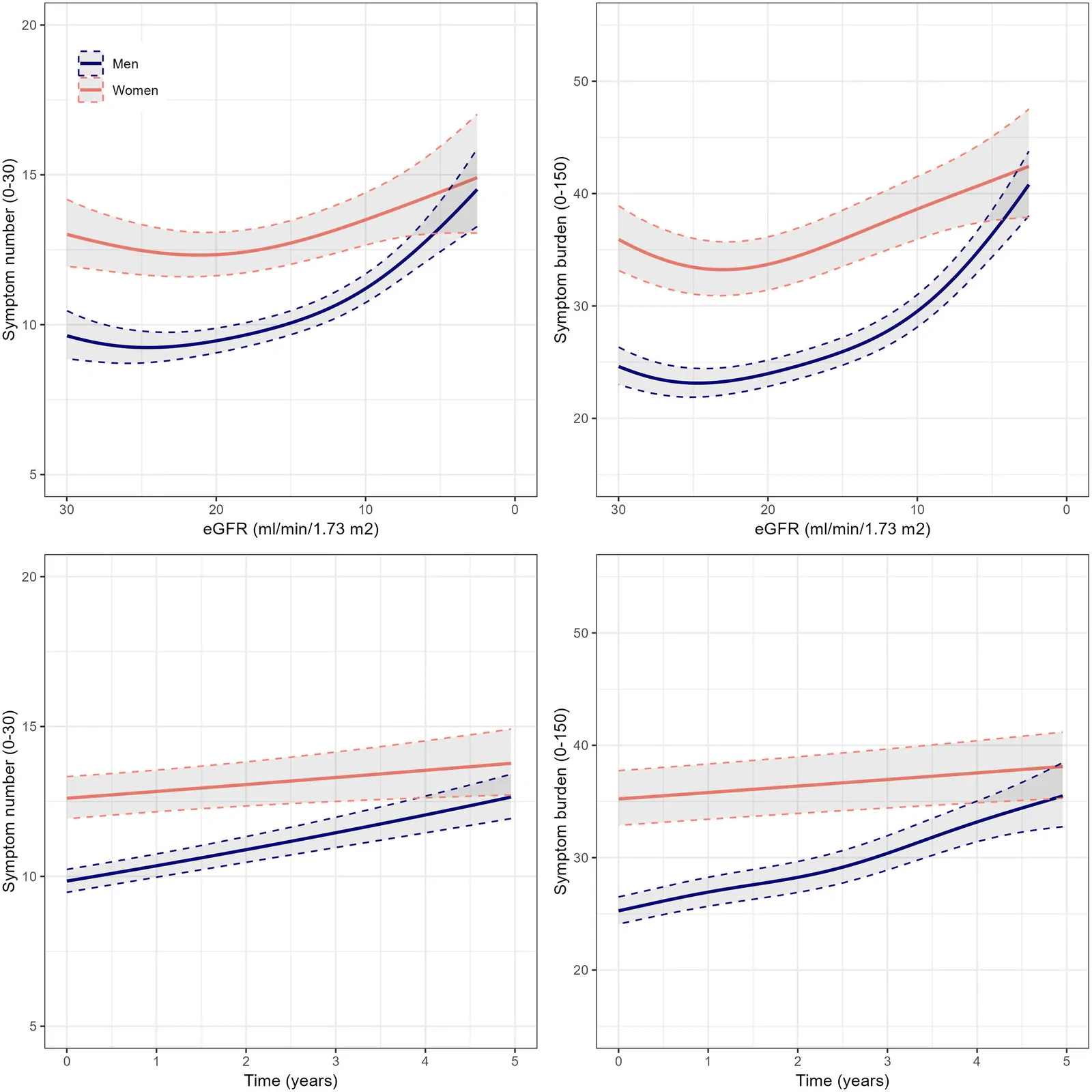
The mean population trajectory for symptom number (ranging from 0 to 30) and symptom severity (ranging from 0 to 150) over eGFR decline and over time, for men and women.

**Table 2:** The average change in overall symptom number and burden for every 5 ml/min/1.73 m^2^ decrease in eGFR by sex.

		Men	Women	Difference			
	Model	Slope	*P* value	Slope	*P* value	Difference	*P* value
Symptom count	Unadjusted	0.97 (0.78–1.16)	<.001	0.34 (0.03–0.64)	.029	0.63 (0.28–0.99)	<.001
	Model 1	1.00 (0.81–1.18)	<.001	0.33 (0.01–0.64)	.040	0.67 (0.30–1.03)	<.001
	Model 2	1.01 (0.82–1.21)	<.001	0.31 (0.00–0.62)	.053	0.70 (0.34–1.07)	<.001
	Model 3	1.01 (0.82–1.21)	<.001	0.37 (0.05–0.68)	.023	0.65 (0.28–1.02)	<.001
Symptom burden	Unadjusted	3.35 (3.01–3.69)	<.001	1.53 (0.97–2.10)	.000	1.82 (1.16–2.47)	<.001
	Model 1	3.40 (3.06–3.74)	<.001	1.42 (0.83–2.00)	.000	1.99 (1.31–2.66)	<.001
	Model 2	3.46 (3.11–3.81)	<.001	1.38 (0.79–1.97)	.000	2.08 (1.39–2.76)	<.001
	Model 3	3.46 (3.11–3.81)	<.001	1.62 (1.03–2.20)	.000	1.84 (1.16–2.53)	<.001

Model 1 adjusts for patient age, marital status, educational class, PKD category, and country. Model 2 adjusts for the variables listed in model 1, as well as smoking status, blood pressure, and BMI. Model 3 adjusts for the variables listed in model 2, as well as Charlson comorbidity score.

**Table 3:** The average change in overall symptom number and burden for every additional year follow-up by sex.

		Men	Women	Difference			
	Model	Slope	*P* value	Slope	*P* value	Difference	*P* value
Symptom count	Unadjusted	0.58 (0.45–0.71)	<.001	0.24 (0.05–0.44)	.016	−0.34 (− 0.57– − 0.10)	.005
	Model 1	0.52 (0.36–0.67)	<.001	0.22 (0.02–0.43)	.034	− 0.30 ( − 0.55– − 0.04)	.024
	Model 2	0.54 (0.39–0.69)	<.001	0.12 ( − 0.09–0.33)	.262	− 0.42 ( − 0.68– − 0.16)	.002
	Model 3	0.53 (0.39–0.68)	<.001	0.15 ( − 0.06–0.36)	.170	− 0.39 ( − 0.64– − 0.13)	.003
Symptom burden	Unadjusted	1.96 (1.71–2.21)	<.001	0.64 (0.29–0.99)	<.001	− 1.32 ( − 1.75– − 0.89)	<.001
	Model 1	2.06 (1.79–2.34)	<.001	0.74 (0.34–1.14)	<.001	− 1.32 ( − 1.81– − 0.83)	<0.001
	Model 2	2.08 (1.80–2.36)	<.001	0.25 ( − 0.11–0.62)	.175	− 1.82 ( − 2.28– − 1.36)	<.001
	Model 3	2.06 (1.78–2.34)	<.001	0.35 ( − 0.02–0.72)	.064	− 1.71 ( − 2.18– − 1.25)	<.001

Model 1 adjusts for patient age, marital status, educational class, PKD category, and country. Model 2 adjusts for the variables listed in model 1, as well as smoking status, blood pressure, and BMI. Model 3 adjusts for the variables listed in model 2, as well as Charlson comorbidity score.

### The evolution of symptom number and severity over eGFR decline

Overall, the number of symptoms increased on average by 0.75 (95% CI 0.58–0.91) symptoms per 5 ml/min/1.73 m^2^ decrease in kidney function, and mean symptom severity increased by 2.79 (95% CI 2.50–3.08) points. The increase in symptom number with declining kidney function was almost three times greater in men (0.97 per 5 ml/min/1.73 m^2^, 95% CI 0.78–1.16) than in women (0.34 per 5 ml/min/1.73 m^2^, 95% CI 0.03–0.64), with similar results found for symptom severity (men: 3.35 points per 5 ml/min/1.73 m^2^, 95% CI 3.01–3.69, women: 1.53 points per 5 ml/min/1.73 m^2^, 95% CI 0.97–2.10). The increase in symptom burden with decreasing kidney function was nonlinear (Fig. 2), with a steepening of symptom number and severity with worsening eGFR, especially in men towards lower levels of eGFR. Adjustment had little effect on the difference in rate of increase between men and women (Table 2). The prevalence of individual symptoms over eGFR decline are presented in Supplemental Fig. 1. The trajectories were heterogenous for different symptoms, but the pattern of a higher prevalence of symptoms in women was present for all symptoms except sexual dysfunction, which was more prevalent in men.

## DISCUSSION

In our European cohort of non-dialysis CKD patients over the age of 65, we demonstrate the longitudinal interdependence of symptom burden, patient gender, and kidney function decline. Over time and in relation to kidney function, different symptoms display heterogeneous trajectories. Overall symptom progression was relatively slow. Although the effect of decreasing eGFR steepened towards lower levels of eGFR (below 10 ml/min/1.73 m^2^), the relative changes per eGFR decline were small. Similar to what is seen in the general population and in other chronic disease populations, including CKD [3, 14–16], we confirm that women report more symptoms and an overall higher symptom burden over the course of disease progression. However, despite women reporting an overall higher symptom burden, the increase over time was more than twice as fast in men during follow-up, and almost three times faster in men when evaluated over eGFR decline.

Several cross-sectional studies in CKD patients demonstrate a high symptom burden in non-dialysis CKD patients. However, the association between symptoms and eGFR has not been clear [4, 17]. On the other hand, longitudinal studies describing the evolution of symptoms in CKD are scarce. Here we demonstrate a nonlinear increase in symptom burden over time and eGFR, especially towards lower levels of eGFR. The CRIC study is one of few other studies assessing symptom development over eGFR decline in 3685 CKD patients not on dialysis, of which 19% had an eGFR <30 ml/min/1.73 m^2^ [18]. Our study expands on their findings by including older patients with eGFR <20 ml/min/1.73 m^2^. In line with our findings, the CRIC authors found a nonlinear association between eGFR and uremic symptom severity score, with a steepening of symptom burden with worsening eGFR. Another similarity was found in the size of effect, with relatively large declines in eGFR showing only small changes in symptom score; a 5 ml/min per 1.73 m^2^ decrease in eGFR resulted in a less than two-point worsening in symptom score on a scale from 0 to 100. Similarly, in our study, a 5 ml/min 1.73 m^2^ decrease in eGFR led to an average increase of 0.75 symptoms on a scale from 0 to 30, suggesting that other factors besides eGFR may be driving symptom burden.

Other longitudinal symptoms studies have focused on the identification of groups of patients with distinct symptom score trajectories (using latent class modelling). Using the Kidney Disease Quality of Life-36 (KDQOL-36) questionnaire, CKD-REIN identified two profiles of symptom trajectories: one-third of participants were characterized by a low initial symptom score that worsened over time, and the remaining two-thirds by a high initial score that remained stable. The former group had more risk factors for CKD progression, a worse health-related quality of life (HRQOL), and a higher risk of kidney replacement therapy and death. Both groups had approximately the same gender distribution [19]. The CRIC study also applied an exploratory clustering approach based on PROMS over time to determine patient groups. Although groups exhibited large differences in baseline scores, trajectories were more or less stable over time [17]. Murtagh *et al*. studied symptom trajectories in CKD stage 5 patients managed conservatively, identifying considerable individual variation in individual trajectories, classifying them as stable, increasing, or fluctuant [20].

Routine assessment of symptoms and symptom burden has previously been shown to help patients maintain or improve quality of life and well-being [21]. To the best of our knowledge, validated PROMs such as the dialysis symptoms index are seldom routinely used for clinical decision making, on for example the timing of dialysis initiation. Given the wide range of uremic symptoms and their inter-individual differences, it is evident that obtaining accurate information from patients about their overall symptom burden during a routine clinical visit is challenging; some degree of information bias is likely to occur, which could potentially influence the timing of dialysis initiation. This argues for the structural and systematic evaluation of symptom burden, at least when eGFR drops below 15 ml/min/1.73 m^2^ and symptoms increase the most. While the adoption of symptoms PROMs is essential, practical barriers remain, including the logistics and routines involved of collecting this information before clinical visits, and transforming it to clinically actionable insights.

In the general population, women tend to report more symptoms than men and consider them to be more severe, even after excluding gynaecological and reproductive symptoms, and after restriction to medically unexplained symptoms [14]. Differences between the sexes in the epidemiology of CKD are well recognized in the field of nephrology, and are also apparent in the patient-reported outcomes literature [22, 23]. Among patients with CKD, cross-sectional studies in both non-dialysis-dependent CKD and in patients on dialysis show that women report a worse mental and physical HRQOL, more symptoms than men, and find symptoms to be more intrusive [24–27]. In the EQUAL cohort, we previously demonstrated a higher symptom number and burden in women at inclusion [28]. We have also reported on the interdependence between symptoms, eGFR, and HRQOL, finding a positive relationship between symptom burden and HRQOL [29], and between eGFR levels and overall symptoms [30]. In this study, we also show that women, on average, report more symptoms and a worse symptom burden than men over time and over kidney decline.

Several mechanisms may be responsible for the overall higher symptom burden reported by women. These differences may be attributed to both biological or physiological differences between males and females (i.e. ‘sex’) or due to culturally and socially constructed roles and characteristics of men and women (‘gender’). As an example, men and women use different strategies to cope with CKD that may affect their symptom burden [31, 32]. Also, men may perceive themselves as better able to cope with the burden of their disease [33], and may therefore be more likely to under-report (physical) weakness [14, 34]. Societal gender stereotypes may play a role here, as women may typically be assumed to be more expressive (about their discomfort), whereas men are conditioned to be tough and that expressing discomfort from symptoms is a sign of weakness. Third, women have a greater incidence of anxiety and depression [35, 36], which may contribute to both physical and mental symptoms, and has been suggested to explain part of the gender difference in HRQOL in patients receiving haemodialysis treatment [37]. Last, chronic pain conditions are more commonly reported by women, and both rodent and human studies suggest that women have lower pain thresholds and tolerance [38]. Evidence suggests these differences arise from both sex-related factors, ranging from genetic, physiological, neuronal, and hormonal differences, as well as gender-related psychosocial differences between men and women [39].

Little is known about the evolution of symptoms specifically in men and women with CKD, and to our knowledge, ours is the first to demonstrate the faster increase in symptom burden over time and over kidney decline in men compared with women. These results are in line with previous findings in the EQUAL cohort; we previously demonstrated that in the year preceding dialysis, HRQOL in men declined at a faster rate compared with women, whereas this decline stabilized more in women than men in the year after dialysis initiation [40]. Similarly, although symptom number and burden worsened considerably before dialysis initiation, in the year after dialysis, the reduction in symptom number and burden was approximately twice as large in women as in men [41]. The mechanisms underlying the faster rate of symptom burden progression in men are difficult to pinpoint. In EQUAL, men have a higher prevalence of comorbidities at inclusion which could affect the speed of symptom progression, however, accounting for these factors through covariate adjustment had little impact on our estimates. It also contradicts the overall higher symptom burden reported by women. Kidney decline is faster in men in the general population, as well as in CKD populations. We previously demonstrated that declines in eGFR were relatively more detrimental to HRQOL in men than in women, proposing that the faster eGFR decline in men and subsequent deterioration in health (or vice versa) leave men with less time to adapt and cope with the imposed physical and mental limitations of CKD [42].

The main strength of our study is that the EQUAL cohort includes a representative sample of carefully phenotyped patients with advanced CKD under nephrology care, from six European countries, with repeated PROMs data and long-term follow-up, making the results generalizable to the clinical practice of European nephrology care. Patients in our study were included when their eGFR dropped below the pre-defined level of 20 ml/min/1.73 m², thus minimizing the risk of survivor bias. Our study has also various limitations that should be addressed. We were probably unable to capture the complex interplay between biological sex-related factors and psychosocial gender-related factors not collected by our study that are also likely responsible for sex differences and, therefore, as in all observational studies, we cannot confer causality. We were able to include most participants in the EQUAL cohort, however, the sample size was reduced due to missing questionnaires during follow-up, although this may be expected given the health status and age of the population [43]. Consequently, this also may have led to some degree of selection bias. Last, as eGFR is a poor indicator of kidney function at lower levels, the relationship between eGFR decline and symptom trajectory should be interpreted with caution when values are low.

In summary, in our European cohort of older patients with advanced CKD, we demonstrate an overall high symptom prevalence and burden, especially in women, but a relatively slow progression of symptoms over time and eGFR. However, despite women reporting an overall higher symptom burden, the increase in symptom burden over time was twice as fast in men during follow-up, and almost three times faster in men when evaluated over eGFR decline. Individual symptom profiles are heterogeneous, which emphasizes the importance of measuring symptom burden using PROMs in routine nephrology care to help identify therapeutic targets and personalize treatment approaches. Further research should focus on how PROMs can routinely be implemented in nephrology care, and how to use PROMs effectively in patient–nephrologist communication with regards to shared decision making.

## Supplementary Material

gfag045_Supplemental_File

## Data Availability

The data used for this publication could be made available at reasonable request to the corresponding authors provided that all legal obligations are fulfilled.

## Notes

12 Supplemental file for details
